# Lack of immunogenicity of xenogeneic DNA from porcine biomaterials^☆^^☆☆^

**DOI:** 10.1016/j.sopen.2022.07.005

**Published:** 2022-07-22

**Authors:** Rae D. Record Ritchie, Sharon L. Salmon, Michael C. Hiles, Dennis W. Metzger

**Affiliations:** a Cook Biotech, Inc, 1425 Innovation Place, West Lafayette, IN, USA 47906; b Department of Immunology and Microbial Disease, Albany Medical College, 47 New Scotland Ave, MC-151, Albany, NY, USA 12208-3479

## Abstract

**Background:**

Clinically useful biomaterials are derived from xenogeneic extracellular matrices, but extensive processes often used to remove all residual DNA are detrimental to their proper biological function. We hypothesized that deliberate and repeated injection of DNA extracted from clinically implantable, xenogeneic extracellular matrices might elicit an immune response in a well-established murine model that could ultimately lead to altered extracellular matrix remodeling.

**Methods:**

DNA was purified from unprocessed porcine extracellular matrices and processed extracellular matrices before sterilization (aseptic) and after sterilization. Groups of 10 mice were injected with these 3 purified DNAs and 3 controls: (1) DNA from *E. coli*; (2) DNA from unprocessed porcine extracellular matrices combined with interleukin-12 and methylated bovine serum albumin and emulsified in incomplete Freund's adjuvant; and (3) buffered saline. Immunizations occurred every 2 weeks for a total of 3 injections. Local cytokines and systemic anti-DNA antibodies were quantified 3 and 7 days after final injection.

**Results:**

The DNA extracted from unprocessed, aseptic, or sterilized porcine extracellular matrices failed to elicit a rejection response, and only with significant, proinflammatory adjuvant activation could such a response be seen. Without the adjuvants, biomaterial-derived DNA resulted in a mild accommodation cytokine response locally and no systemic anti-DNA antibody expression even at doses approximately 100-fold larger than would be clinically likely via extracellular matrix implantation.

**Conclusion:**

The immunological safety of porcine extracellular matrix biomaterials appears not to be related to DNA residues present. Such biomaterials need not be extensively processed, likely leading to detrimental changes in their bioactivity, solely in an effort to remove the mammalian DNA.

## Highlights

• Xenogeneic DNA from decellularized biomaterial does not elicit an immune response.
• Such materials need not be extensively processed solely to remove the remnant DNA.

## INTRODUCTION

DNA codifies the instructions of life. Because we constantly are bombarded with it from many sources, including eating it daily, it makes teleological sense that DNA would not be a source of immune stimulation or rejection—yet little has been published to investigate this. Biologic devices derived from extracellular matrices (ECMs) are commercially available globally to treat a variety of soft-tissue injuries. Most are manufactured by decellularizing ECMs of different organs, and the multiple processing techniques used result in varying degrees of cellular remnants. The amount of cellular debris has been linked to a host response in animal models [1]. Some have suggested that the amount of donor DNA present in ECM is responsible for a poor response [2], yet there is no linkage between the presence of residual DNA and successful patient outcome [3].

For decades, it has been well established that nucleic acids (both DNA and RNA) are not efficient as transfectable vectors without capsids, liposomes, or other delivery mechanisms that protect them from degradation and aid in their access to the cellular cytoplasm. Thus, naked DNA does not appear to be a significant source of disease transmission risk, but is it a source of aberrant inflammation when present during wound healing? A very large body of data from millions of implants of allogeneic and xenogeneic origin suggests that some cellular remnants, especially those containing high concentrations of cell-surface antigens, may elicit limited immune responses, but nothing directly implicates DNA as an immunogen. In fact, DNA vaccines composed of DNA alone do not induce anti-DNA antibodies [4,5]. Teleologically, a nonantigenic property of DNA makes sense because it is ubiquitous. An unproven hypothesis exists in medicine that the presence of residual DNA in decellularized ECM evokes an unwanted immune response. This assertion has been especially repeated in the field of orthopedics [[6], [7], [8]], but no direct association has been shown. Therefore, we chose to test this hypothesis, ie*,* that in vivo inoculation of xenogeneic DNA fragments could stimulate a measurable, host immune response, and we chose to test levels of DNA immunization many folds greater than would be expected to be a clinical exposure.

We used the small intestinal submucosa (SIS) as the model ECM. SIS has been used in commercially available products for more than 20 years in millions of patients and is the subject of over a thousand peer-reviewed publications. In addition, there are other, similar submucosa/ECM materials used for medical devices. Here we demonstrate that neither full-length mammalian dsDNA from unprocessed SIS nor smaller dsDNA remnants harvested from aseptic or sterilized ECM (SIS) were able to induce an anti-DNA antibody response without the addition of strong co-stimulators, incomplete Freund's adjuvant (IFA), methylated bovine serum albumin (mBSA), and interleukin-12 (IL-12). Further, as previously shown for decellularized ECM [9], no Th1-associated cytokines were induced upon immunization with the extracted dsDNA from sterilized (clinical-grade) ECM.

## MATERIALS AND METHODS

### Mice

BALB/c mice (female, 6–8 weeks, 18–20 g) were obtained from Jackson Laboratories (Bar Harbor, ME). All mice were maintained under specific pathogen-free conditions with individually ventilated cages in the Animal Research Facility at Albany Medical College. Female mice were used for their more robust antibody response and increased likelihood to produce anti-DNA antibodies (eg, in systemic lupus erythematosus) [10] as well as for continuity with previous studies using decellularized ECM [9].

Experimental animal protocols were in accordance with the *Guide for the Care and Use of the Laboratory Animals* of the NIH [11]. All animal procedures were approved by the Institutional Animal Care and Use Committee at Albany Medical College (protocol number: 20-04001). There were no other criteria set for including or excluding animals (or data points). The mice were randomized into separate cages upon arrival at Albany Medical College. There was no strategy to minimize confounders. Both DWM and SLS were aware of the group allocation at all stages of the experiment.

### Source Tissue (ECM)

The source material, porcine small intestine, was obtained from Cook Biotech, Inc, and treated according to Cook Biotech, Inc, standard manufacturing protocols. SISs from three different steps of manufacturing were used as described in Table 1. In the initial step, the submucosa of porcine small intestine was mechanically separated from other layers of the intestine, rinsed in water, and frozen at − 80°C until processed. In the second step, thawed SIS was disinfected and decellularized via the commonly used peracetic acid and ethanol solution followed by high-purity water rinses and finally lyophilized into sheets. The third step was terminal sterilization by ethylene oxide gas under standard temperatures, pressures, and durations.

**Table 1:** Description of the steps of production of the SIS that was used for extracting DNA⁎

*Step*	*Description*	*Abbreviated description*
Step 1	SIS mechanically separated from the other layers of the small intestine	Unprocessed ECM
Step 2	After step 1, decellularized, disinfected, and then lyophilized	Aseptic ECM
Step 3	After step 2, terminally sterilized with ethylene oxide	Sterilized ECM

⁎ Details of the material processing can be found in several patents including US Patent #6,206,931.

### Other Reagents

Deoxyribonucleic acid, sodium salt, from *Escherichia coli* strain B; calf thymus DNA; incomplete Freund's adjuvant (IFA); proteinase K; lysozyme; and methylated BSA (mBSA) were purchased from Sigma-Aldrich (St Louis, MO). Novagen pig genomic DNA was purchased from MilliporeSigma (St Louis, MO). Genomic Maxi-tips, Genomic DNA Buffer sets, and RNase A were purchased from Qiagen (Germantown, MD). TE buffer, pH 8.0, was purchased from Amresco Life Science/VWR (Radnor, PA). A 200-bp DNA ladder (New England Biolabs), 96-well Maxisorp microplates, and T-PER (Tissue Protein Extraction Reagent) were purchased from New Thermo Scientific (Waltham, MA). Goat anti-mouse IgG (H + L) conjugated to horseradish peroxidase was purchased from Novus Biologicals (Littleton, CO). TMB substrate (BD OptEIA Reagent) was purchased from BD Biosciences (San Jose, CA). A ProcartaPlex Mouse Cytokine/Chemokine Panel 1A 36plex Kit was purchased from Invitrogen (Carlsbad, CA).

### Isolation of Porcine DNA (pDNA)

DNA was isolated from tissue using Genomic Maxi-tips, Genomic DNA Buffer sets, RNase A, proteinase K, and lysozyme. In general, the Qiagen Genomic DNA Handbook was followed.

SISs from three different steps of manufacturing, as described in Table 1, were cut into small pieces, weighed (< 0.85 g/tube), and placed into 50-mL tubes. RNase A in Buffer G2 was added followed by proteinase K (final concentration 1 mg/mL). Samples were placed on a 56°C heat block and vortexed intermittently until the tissue was dissolved. Eluted DNAs were precipitated with isopropanol and centrifuged at 10,000*g* for 15 minutes at 4°C. The supernatants were discarded, and the pellets were washed with cold 70% ethanol and centrifuged again. The supernatants were discarded, and the pellets were air-dried. The pellets and the *E. coli* DNA were resuspended in TE buffer, pH 8.0, in a 37°C incubator. The concentrations of the solutions were determined spectrophotometrically at 260 nm, and purity was determined by 260/280 ratios using a FLUOstar Omega microplate reader (BMG Labtech, Cary, NC).

DNAs were concentrated by the addition of 1/10th vol of 3 M sodium acetate, pH 5.2, followed by 2–2.5 × vol of ice-cold 100% ethanol and placing at − 80°C for 15 min. The solutions were centrifuged at 10,000*g* for 15 minutes at 4°C, the supernatants were discarded, and the pellets were washed with 95% ethanol and centrifuged again. The pellets were then air-dried. High-purity water was added to resuspend the pellets at approximately 1 mg/mL, and the solutions were placed in a 37°C incubator to dissolve. The concentration of the solutions was determined spectrophotometrically at 260 nm, and purity was determined by 260/280 ratios using a FLUOstar Omega microplate reader (BMG Labtech, Cary, NC).

**Supplemental Fig 1:**
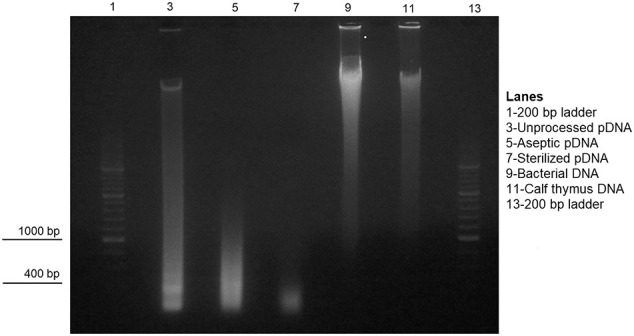
Agarose gel analysis with EtBr for visualization of the DNA samples used in the study. Each lane contained 6 μg of DNA. A 200-bp DNA ladder was used to estimate size, and a sample of calf thymus DNA was used as a control for full-length mammalian DNA.

The solutions were electrophoresed on a 0.8% agarose gel in TAE buffer with EtBr for visualization (Supplemental Fig 1). Each lane contained of 6 μg of DNA; a 200-bp DNA ladder was used to estimate size, and a sample of calf thymus DNA was run as a control.

The solutions were lyophilized overnight and, after resuspension in phosphate-buffered saline (PBS), were used for injections and for coating ELISA plates for determination of antibody titers.

### Immunization

Groups of 10 BALB/c mice were anesthetized with isoflurane and injected intramuscularly with one of the solutions described in Table 2. Immunizations were performed every 2 weeks for 3 total injections per mouse. Five (5) mice per group [12] were sacrificed 3 days after the final injection, and cytokine expression was measured in the thigh biopsy. The remaining mice were bled 1 week after the last injection to measure serum anti-DNA antibodies.

**Table 2:** Immunization groups

*Group type*	*Description*	*Abbreviated description*
Experimental	50 μg of pDNA from unprocessed SIS in 50 μL PBS	Unprocessed pDNA
Experimental	50 μg of pDNA from aseptic SIS in 50 μL PBS	Aseptic pDNA
Experimental	50 μg of pDNA from sterile SIS in 50 μL PBS	Sterile pDNA⁎
Control	50 μg of pDNA from unprocessed SIS mixed with mBSA (75 μg/mouse) and murine rIL-12 (1 μg/mouse) [9,10] followed by emulsification in incomplete Freund's adjuvant	Unprocessed pDNA with mBSA, IL-12, emulsified in IFA
Control	50 μg of commercially available bacterial DNA in 50 μL of PBS	Bacterial DNA
Control	50 μL PBS alone	PBS

⁎ Three doses of 50 μg of pDNA in a mouse corresponds to ~ 100-fold greater DNA exposure than would be seen by implantation of one of the largest clinical ECM grafts in a 60-kg person.

### Analysis of Cytokine Expression

Three days after the final injection, the site of injection was biopsied and weighed, and a tissue homogenate was prepared using a gentleMACS OctoDissociator (Miltenyi Biotec, Inc, Bergisch Gladbach, Germany). Each homogenate was placed in 600 μL of T-PER (Tissue Protein Extraction Reagent) and then aliquoted and stored at − 80°C until analysis. Levels of IFN-γ, IL-4, IL-5, TNF-α, IL-1α, and IL-1β protein were determined using the ProcartaPlex Mouse Cytokine/Chemokine Panel 1A 36plex Kit and a Bio-Plex 200 System (Bio-Rad Laboratories, Hercules, CA). One (1) sample was lost from the aseptic pDNA group, resulting in *n* = 4 for this group; all other groups have *n* = 5.

### ELISA for Anti-DNA Antibody

Sera were collected 1 week after the final injection, and anti-DNA antibody levels were measured by ELISA. Briefly, 96-well Maxisorp microplates were coated with 5 μg/mL DNA overnight at 4°C. The plates were then washed with PBS containing 0.05% Tween-20 (PBS-T). Serial dilutions of sera in PBS-T were added to the wells and incubated at room temperature for 2 hours. The plates were washed with PBS-T followed by the addition of goat anti-mouse IgG (H + L) conjugated to horseradish peroxidase for 90 minutes. The plates were again washed with PBS-T followed by the addition of TMB substrate (BD OptEIA Reagent). Absorbance was measured at 450 nm using a Power Wave HT microplate reader (BioTek, Winooski, VT). To test for inhibition of reactivity by soluble bacterial DNA, sera were diluted 1:10 in PBS and preincubated for 30 minutes at room temperature with 50 μg/mL of bacterial DNA before addition to DNA-coated plates.

### Statistical Analysis

The data were analyzed using 1-way ANOVA and Tukey–Kramer HSD or Student *t* test.

## RESULTS

The overall goal herein was to model whether DNA fragments that are present within clinical-grade small intestinal submucosa (SIS) could be immunostimulatory. We extracted porcine DNA (pDNA) from unprocessed, aseptic, and sterile SIS (Table 1). Visualization on an agarose gel (Supplemental Fig 1) demonstrated that the size of the pDNA decreased from full length to < 500 bp after processing. We immunized mice with pDNA obtained from unprocessed SIS; small pDNA fragments from aseptic SIS; small pDNA fragments from sterile SIS; full-length pDNA from unprocessed SIS that was emulsified in IFA after admixture with mBSA and IL-12 as co-stimulators [9,10]; bacterial DNA as a control to demonstrate that the mice could produce antibodies; or PBS alone. The mice were immunized at 2-week intervals (three injections total), and expression of immune cytokines at the injection site was then measured.

### Cytokine Induction by SIS-Derived pDNA

**Fig 1:**
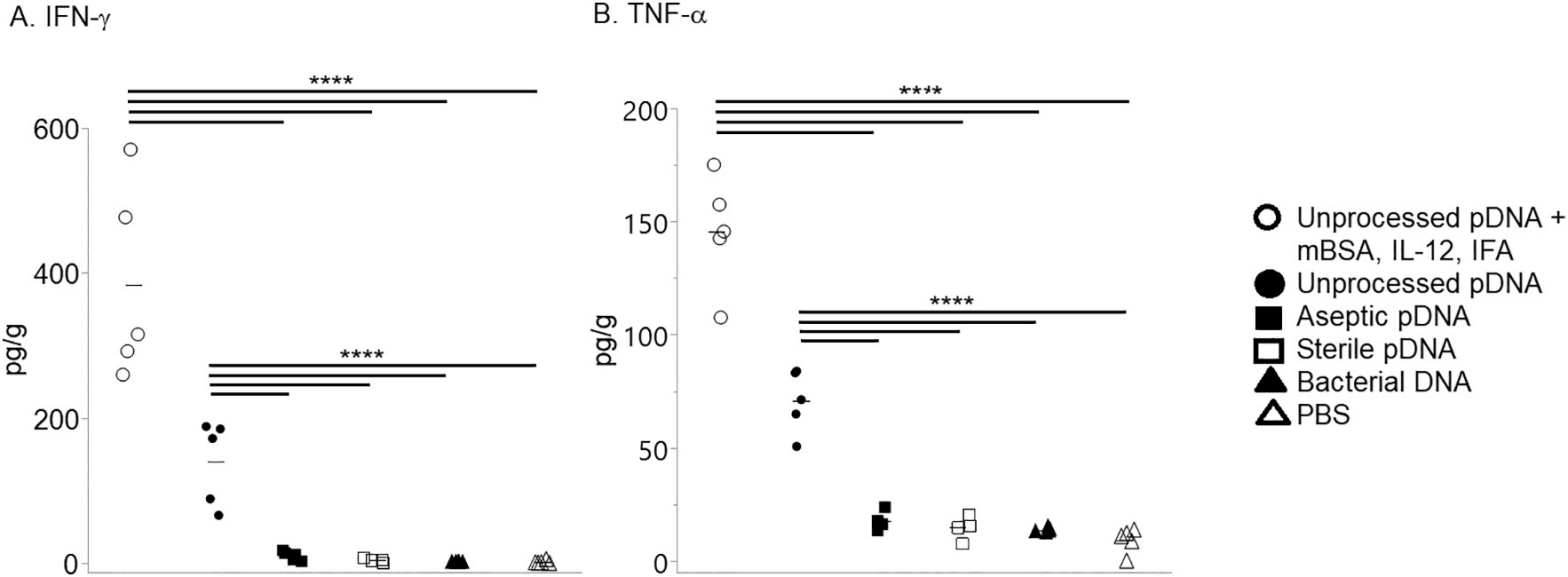
Expression of Th1 cytokines IFN-γ and TNF-α after immunization with DNA. Mice were immunized at 2-week intervals for a total of 3 injections. Three (3) days after the final injection, the injection site was biopsied, and cytokine levels in the tissue homogenates were quantified by multiplex analysis. Each symbol represents results from an individual mouse; 5 mice/group except for aseptic pDNA, 4 mice/group; bars equal mean. The data were analyzed using Tukey–Kramer HSD; ****P < .0001.

**Fig 2:**
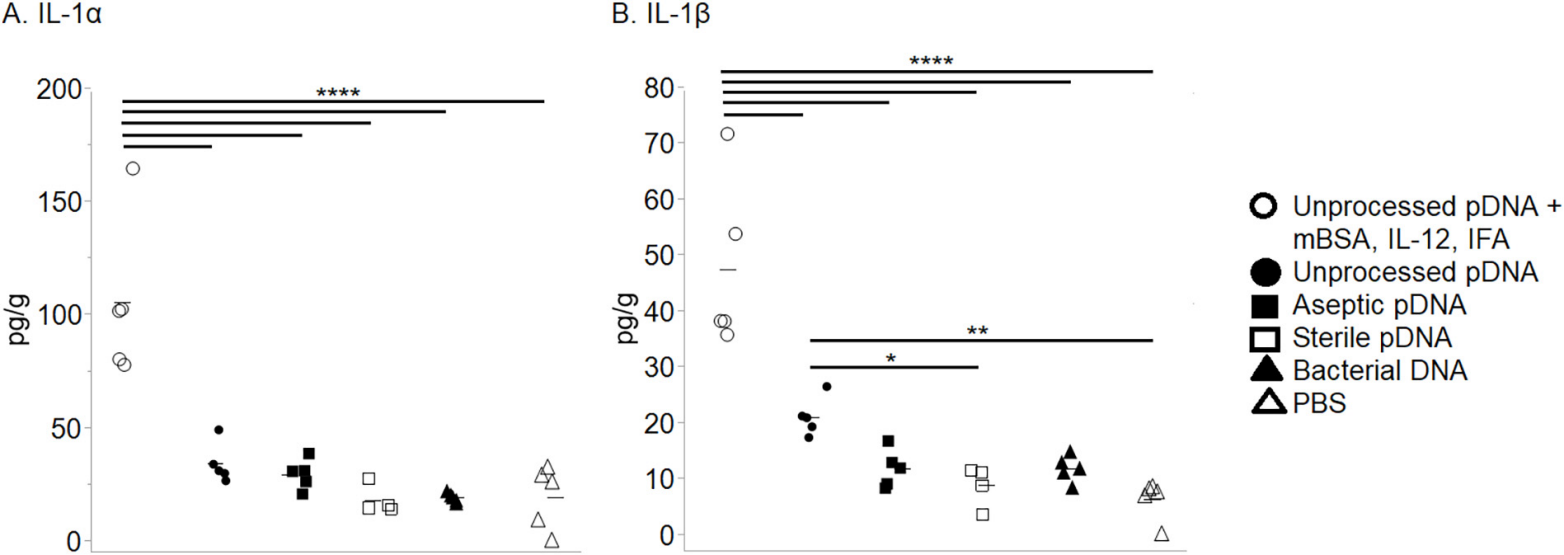
Expression of proinflammatory cytokines IL-1α and IL-1β after immunization with DNA. Mice were immunized at 2-week intervals for a total of 3 injections. Three (3) days after the final injection, the injection site was biopsied, and cytokine levels in the tissue homogenates were quantified by multiplex analysis. Each symbol represents results from an individual mouse; 5 mice/group except for aseptic pDNA, 4 mice/group; bars equal mean. The data were analyzed using Tukey–Kramer HSD; *P < .05, **P < .01, ****P < .0001.

Immunization with methylated-BSA (mBSA) and pDNA derived from unprocessed SIS, under proinflammatory, Th1-inducing conditions, ie, in the presence of IL-12 and IFA, induced high levels of IFN-γ at the injection site that was statistically different from all other groups (Fig 1, *A*). This result agrees with previous reports on the effects of IL-12 [13,14]. This group of animals also expressed the inflammatory cytokines TNF-α, IL-1α, and IL-1β (Figs 1, *B* and 2, *A*-*B*).

**Fig. 3:**
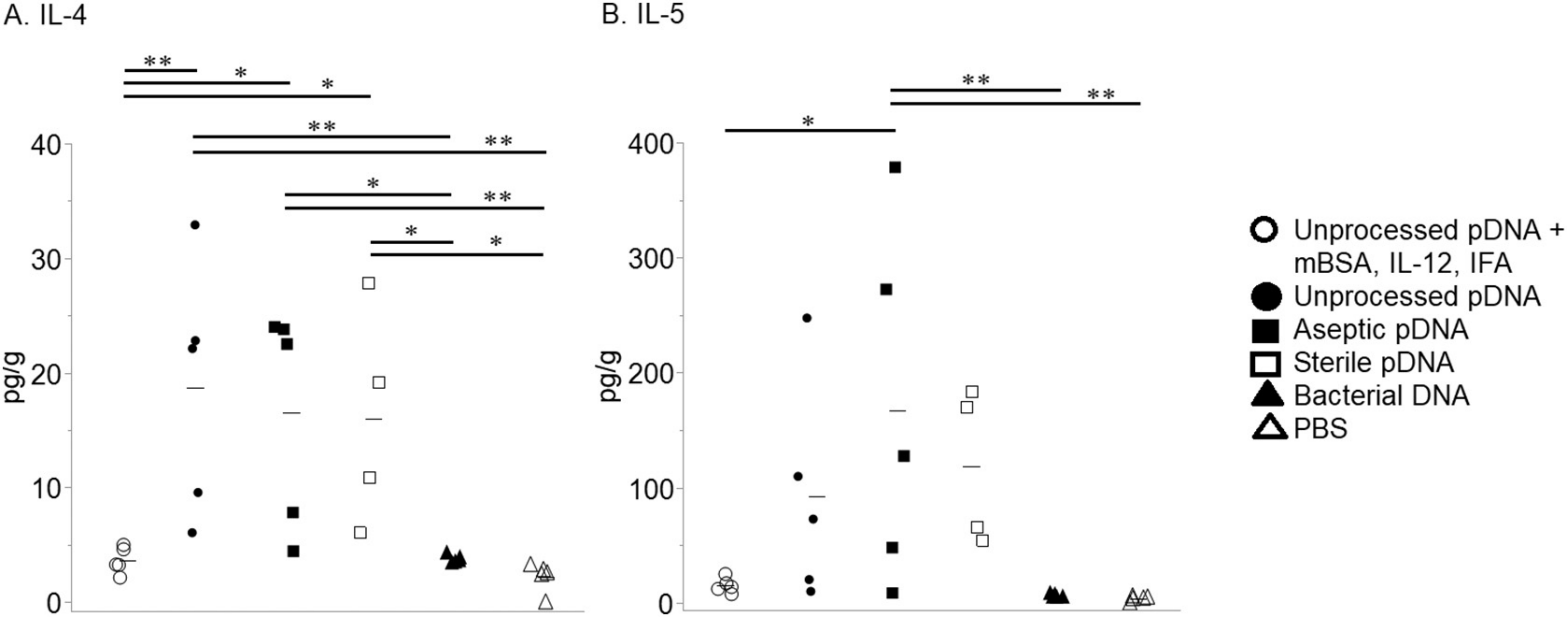
Expression of Th2 cytokines IL-4 and IL-5 after immunization with DNA. Mice were immunized at 2-week intervals for a total of 3 injections. Three (3) days after the final injection, the injection site was biopsied, and cytokine levels in the tissue homogenates were quantified by multiplex analysis. Each symbol represents results from an individual mouse; 5 mice/group except for aseptic pDNA, 4 mice/group; bars equal mean. The data were analyzed using Tukey–Kramer HSD; *P < .05, **P < .01.

Conversely, and in agreement with what is observed following implantation of SIS [9], animals immunized with unprocessed SIS-derived pDNA, in the absence of any additional costimulatory molecules/adjuvants, or with aseptic or sterile SIS-derived pDNA expressed the Th2-associated cytokine IL-4 at levels higher than the costimulatory, bacterial DNA and PBS groups (Fig 3, *A*). In addition, the animals immunized with aseptic SIS-derived pDNA expressed IL-5 at levels higher than the costimulatory, bacterial DNA and PBS groups (Fig 3, *B*).

The animals immunized with unprocessed SIS-derived pDNA (no costimulators) also expressed TNF-α but at levels lower than the group with costimulators (Fig. 1, *B*); more IFN-γ than the aseptic and sterile SIS-derived pDNA, bacterial DNA, and the PBS groups (Fig 1, *A*); and more IL-1β than sterile SIS-derived pDNA and PBS (Fig 2, *B*).

Immunization with bacterial DNA in all cases resulted in cytokine levels that were essentially equivalent to those observed in control mice injected with PBS (Fig 1, Fig 2, Fig. 3).

### pDNA from SIS Failed to Induce Anti-DNA Antibody

**Supplemental Fig 2:**
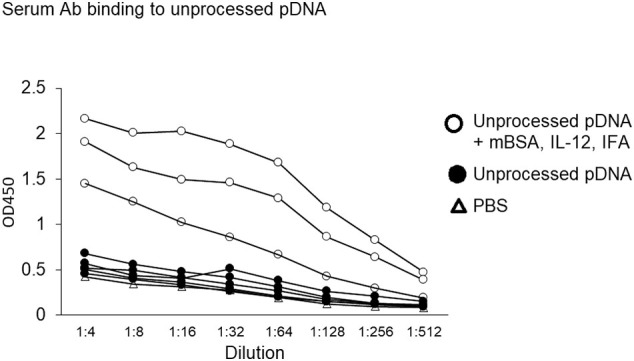
Serum antibody binding to unprocessed pDNA. Mice were immunized with PBS as a control or with unprocessed pDNA with or without mBSA, IL-12, and IFA. Serial dilutions of individual sera were then tested for binding to plates coated with unprocessed pDNA. Each line represents results from an individual mouse; 3–5 mice/group.

ELISAs were next performed to determine expression of serum anti-DNA antibodies following immunization. A titration analysis of sera from individual mice showed that all animals immunized with unprocessed SIS-derived full-length pDNA mixed with mBSA and IL-12 and emulsified in IFA expressed pDNA-binding antibody, whereas mice immunized with unprocessed SIS-derived pDNA in PBS did not (Supplemental Fig 2).

**Fig. 4:**
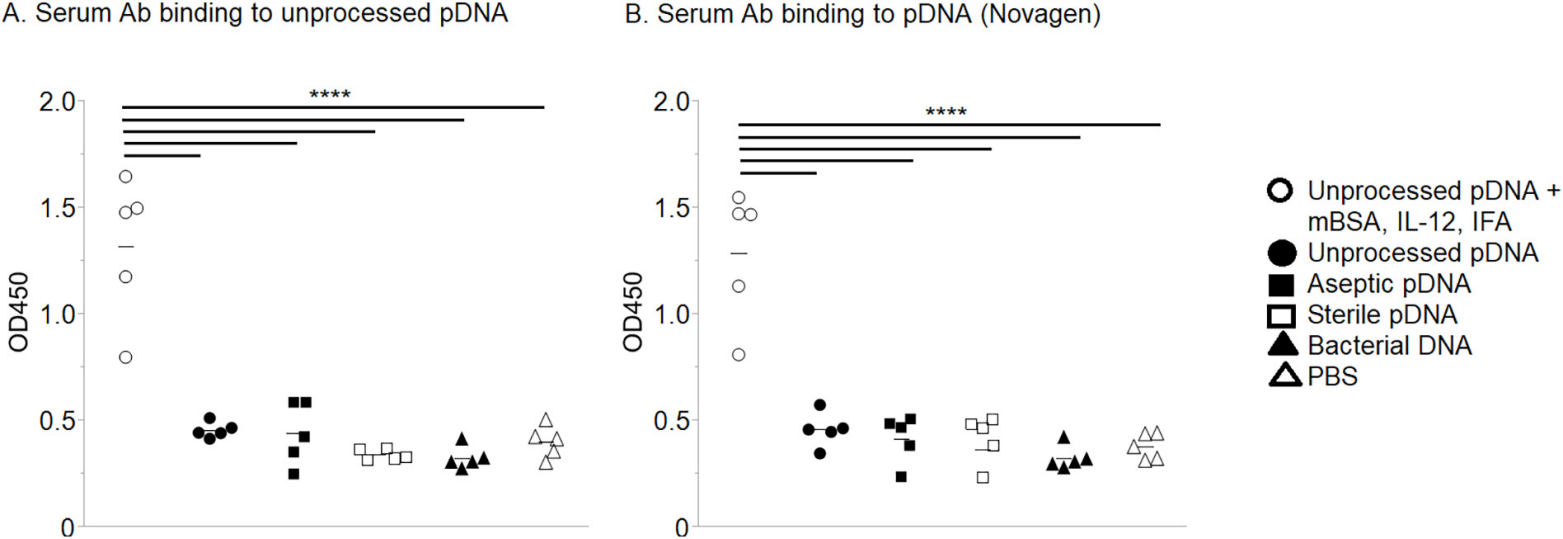
Serum antibodies binding to unprocessed pDNA. Mice were injected with unprocessed pDNA with or without mBSA, IL-12, and IFA or with aseptic pDNA or with sterile pDNA. Additional groups of mice were injected with bacterial DNA or PBS as controls. Individual sera diluted 1:10 in PBS were then tested for binding to plates coated with (A) unprocessed pDNA or (B) Novagen pDNA, purchased commercially. Each symbol represents results from an individual mouse; 5 mice/group. The data were analyzed using Tukey–Kramer HSD; ****P < .0001.

Sterile SIS typically contains small remnants (< 500 bp) of pDNA rather than full-length pDNA, which results from the processing procedure [15]. We considered the possibility that such pDNA remnants could be immunogenic in a manner distinct from full-length pDNA. Nevertheless, binding of sera antibodies to pDNA was observed only when immunization was performed with full-length (unprocessed) pDNA in the presence of mBSA, IL-12, and IFA (Fig 4, *A*). Antisera obtained following immunization with unprocessed SIS-derived full-length pDNA in PBS or after immunization with aseptic or sterile SIS-derived pDNA remnants, containing potentially unique epitopes, failed to induce reactivity to unprocessed SIS-derived full-length pDNA (Fig 4, *A*). Neither full-length, double-stranded pDNA nor remnants below 500 bp in length, in levels of DNA many folds greater than would be expected in a clinical exposure, caused mice to produce anti-pDNA antibodies.

We also considered the possibility that SIS-derived DNA could be contaminated with bacterial DNA from the intestinal microbiota. However, the induction of anti-bacterial DNA antibodies was unlikely because no reactivity to unprocessed SIS-derived full-length pDNA was observed by antisera from any pDNA (without co-stimulants) immunized mice (Fig 4, *A*). Furthermore, immunization with bacterial DNA failed to induce antibody that bound to unprocessed SIS-derived full-length pDNA. Identical results were obtained using ELISA plates coated with Novagen full-length pDNA purchased commercially (Fig 4, *B*). We conclude that neither SIS-derived pDNA nor bacterial DNA induces antibodies that are reactive with native pDNA.

### Reactivity of Anti-DNA Antibodies to pDNA Fragments From Aseptic or Sterile SIS

**Fig. 5:**
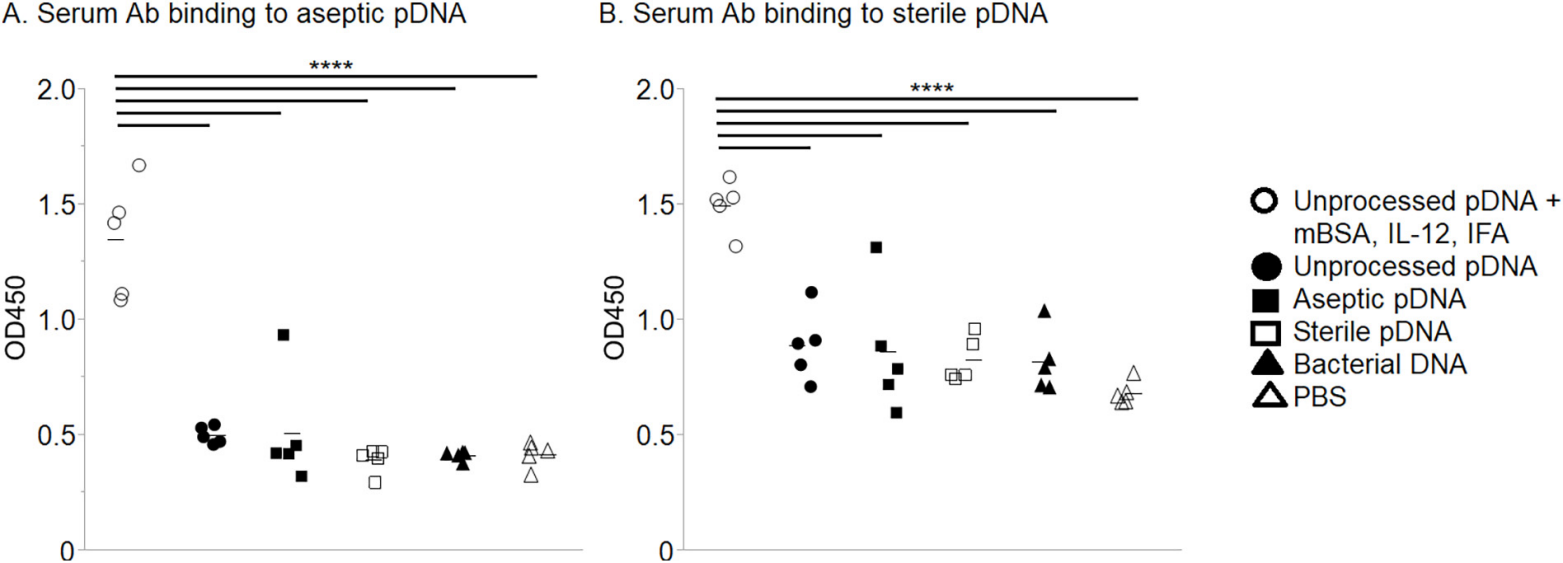
Serum antibodies binding to pDNA fragments. Mice were immunized with unprocessed pDNA, with or without mBSA, IL-12, and IFA; with aseptic pDNA; with sterile pDNA; with bacterial DNA; or with PBS as a control. Individual sera, diluted 1:10 in PBS, were then tested for binding to plates coated with (A) aseptic pDNA or (B) sterile pDNA. Each symbol represents results from an individual mouse; 5 mice/group. The data were analyzed using Tukey–Kramer HSD; ****P < .0001.

We further investigated whether the above immunization procedures might induce antibodies reactive with unique epitopes exposed on small remnants of SIS-derived pDNA. Again, it was observed that detectable reactivity to aseptic or sterile SIS-derived pDNA was seen only with sera obtained from mice immunized with unprocessed SIS-derived full-length pDNA in the presence of mBSA, IL-12, and IFA (Fig 5, *A*–*B*). In every case, immunization with unprocessed SIS-derived full-length pDNA in PBS, < 500 bp remnants from aseptic or sterile SIS, or bacterial DNA failed to induce reactivity to pDNA remnants that were prepared from aseptic SIS (Fig 5, *A*) or sterile SIS (Fig 5, *B*).

### Reactivity of Anti-pDNA Antibodies to Bacterial DNA

**Fig. 6:**
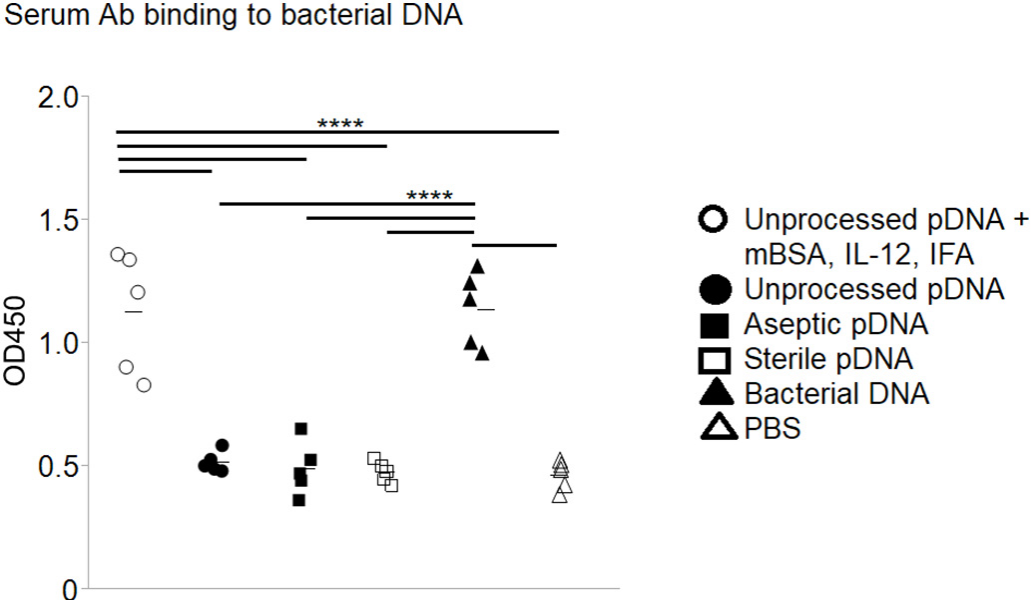
Serum antibodies binding to bacterial DNA. Mice were immunized with unprocessed pDNA, with or without mBSA, IL-12, and IFA; with aseptic pDNA; with sterile pDNA; with bacterial DNA; or with PBS as a control. Individual sera, diluted 1:10 in PBS, were tested for binding to plates coated with bacterial DNA. Each symbol represents results from an individual mouse; 5 mice/group. The data were analyzed using Tukey–Kramer HSD; ****P < .0001.

**Fig. 7:**
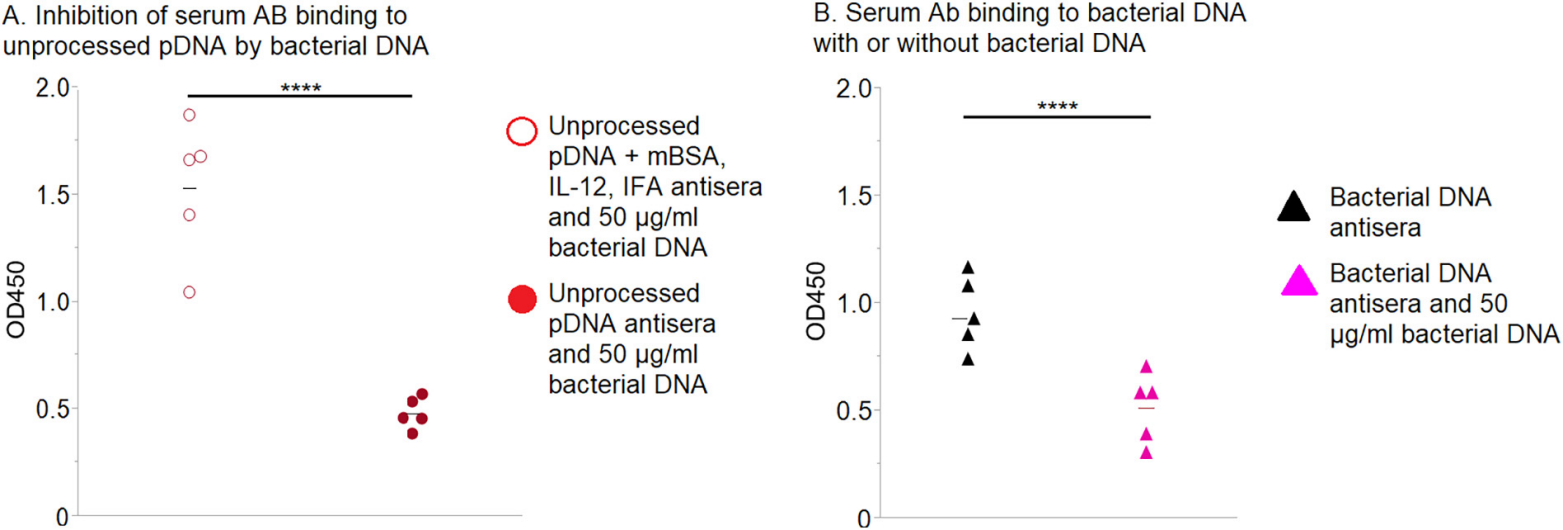
Inhibition of serum antibodies binding by preincubation with bacterial DNA. (A) Mice were immunized with unprocessed pDNA + mBSA, Il-12, and IFA or with unprocessed pDNA. Their antisera, diluted 1:10 in PBS, were preincubated with 50 μg/mL of soluble bacterial DNA and then tested for binding to plates coated with unprocessed pDNA. (B) Mice were immunized with bacterial DNA, and their antisera were diluted 1:10 in PBS. Half of the diluted sera were preincubated with 50 μg/mL of soluble bacterial DNA, and then all sera were tested for binding to bacterial DNA-coated plates. Each symbol represents results from an individual mouse; 5 mice/group. The data were analyzed using Student *t* test; ****P < .0001.

As stated above, it is possible that DNA present within SIS is partially intestinal bacterial DNA. Although we saw no binding of antisera generated by immunization with bacterial DNA to pDNA, it was important to establish our ability to actually generate bacterial DNA-binding antibodies. Thus, animals were immunized with bacterial DNA, and then the antisera were tested for reactivity to bacterial DNA. It was found that serum antibodies from bacterial DNA-immunized animals bound to bacterial DNA coated plates (Fig 6). None of the sera from mice immunized with SIS-derived pDNA reacted with bacterial DNA, except from those animals immunized with unprocessed SIS-derived full-length DNA, mBSA, and IL-12 emulsified in IFA. Presumably, this latter, intense inflammatory scheme generated nonspecific immunoglobulins that could cross-react with porcine and bacterial DNA. Indeed, although anti-pDNA antisera generated using mBSA, IL-12, and IFA bound to bacterial DNA, preincubation of the anti-pDNA antisera with bacterial DNA did not prevent binding to pDNA (Fig 7, *A*). On the other hand, preincubation of antibacterial DNA antisera with soluble bacterial DNA prevented binding to bacterial DNA-coated plates (Fig 7, *B*). We conclude that if bacterial DNA is present in SIS, it does not induce a detectable antibody response.

### Effect of Ethylene Oxide Terminal Sterilization

Ethylene oxide (used as a sterilant) creates DNA adducts [16], and when there are high levels of such DNA modifications, antibodies can be created [17]. To determine if the ethylene oxide used to sterilize SIS changed the antigenicity of the SIS-derived pDNA, the immune response with and without terminal sterilization was determined (aseptic versus sterilized SIS). Whereas sterilization reduced the size of the dsDNA (Supplemental Fig 1), neither double-stranded pDNA from SIS before or after terminal sterilization induced antibodies (Fig. 4, Fig. 5) or Th1 cytokines (Fig 1, Fig 2).

## DISCUSSION

The overall goal herein was to model whether DNA fragments that are present within small intestinal submucosa (SIS) could be immunostimulatory in humans using a mouse model surrogate. Upon implantation in mice, SIS is known to generate a Th2-restricted immune response [9]; this was demonstrated by determining the expression of cytokine mRNA at the implantation site. This same report also immunized mice with an SIS extract (Tris buffer) in Freund's adjuvant and found that anti-SIS antibodies were of both Th1 and Th2 subtypes, whereas anti-SIS antibodies after implantation without Freund's adjuvant were only of the Th2 subtype (IgG1 in mice). This report did not determine the specific antigenic molecule(s) but mentioned unpublished work demonstrating that anti-SIS antibodies reacted with proteinaceous components of SIS. Follow-on work verified that anti-SIS antibodies after SIS implantation are only of the Th2 subtype [18]. Supporting the in vivo studies in mice, in vitro studies with human cells have demonstrated that intact SIS suppress Th1 cell expansion and the secretion of IL-12 and IFN-γ by Th1 cells [19] and do not induce IL-6 or IL-12 secretion by B-cells [20]. Although not measured in the reports, it is assumed that the SIS contained pDNA. In addition, Huleihel et al [21] demonstrated that human THP1 cell gene expression in response to SIS hydrogel exposure was different from classical Th1 and Th2 responses. Again, the amount of DNA in the SIS hydrogel was not determined, but others have shown that digesting ECM into a hydrogel does not remove the DNA [22]. Together, these studies with SIS, presumably containing pDNA, indicate a Th2 response.

In humans treated for chronic venous ulcers with SIS, patients whose wounds healed had a reduction in proinflammatory (Th1) cytokines in wound fluid with an increase in TGF-β compared to before treatment [23]. In agreement, we demonstrated in this study that injection of pDNA remnants derived from aseptic and sterilized SIS did not induce proinflammatory cytokines at the site of injection (Fig 1, Fig 2). This is also in agreement with previous reports that whereas bacterial DNA and CpG ODN induce Th1 cytokines, mammalian DNA does not [24]. In addition, DNA from dead mammalian cells can induce Th2 differentiation in vitro [25]. It is likely that this type of sterile injury, not an acute inflammatory response [26], was induced in the present study. It must also be noted that the pDNA derived from the unprocessed SIS stimulated the production of IFN-γ and TNFα at levels significantly higher than the other groups but lower than the pDNA group with costimulators (Fig 1, *A*–*B*). No attempt was made in this work to separate mitochondrial (mtDNA) from nuclear DNA (nDNA) because both could be expected to be present as cellular remnants. This is important because mtDNA, which is circular and has unmethylated CpG motifs like bacterial DNA, can bind TLR9 [27], resulting in the production of IFN-γ and TNF-α. This could explain the weak Th1 immune response that resulted in the unprocessed SIS-derived pDNA group without costimulators.

In addition to cytokine responses, we determined whether injection of SIS-derived pDNA remnants would stimulate production of anti-pDNA antibodies. It has been documented that implantation of SIS can induce expression of anti-SIS antibodies. In mice, anti-SIS antibodies can be detected after SIS implantation, but only of the Th2 antibody subtype [9,18,28]. In nonhuman primates, only anti-α-Gal antibodies were detected and not anti-SIS antibodies when SIS was implanted in the abdominal wall [29]. Reports of antibody responses in humans are conflicting. Hodde et al [23] found that there was no anti-SIS or anti-α-Gal antibody response when patients were treated repeatedly with SIS for chronic leg ulcers. However, Ansaloni et al [30] reported that all 5 patients followed after inguinal hernioplasty with SIS had detectable anti-SIS and anti-α-Gal serum antibodies, with levels peaking between 2 and 6 weeks after surgery. In both examples [22,30], the ELISA plates were coated with Tris extracts of SIS as the antigen.

In an attempt to determine if the previously reported anti-SIS antibodies might be reactive with pDNA, we injected sterile SIS-derived pDNA remnants into mice. Previous reports have found that mammalian DNA alone does not induce anti-DNA antibodies in normal mammals [12,31] and that plasmid DNA vaccines (no adjuvants) do not produce anti-DNA antibodies in humans [4,5]. There are instances of anti-self-DNA antibody expression resulting from dead cell clearance, but this occurs in certain abnormalities, such as a lack of DNase II [32]. We found that sterile SIS-derived remnant pDNA did not induce an antibody response (Fig. 4, Fig. 5, Fig. 6). In fact, only full-length pDNA with costimulators or bacterial DNA induced anti-DNA antibodies (Fig. 4, Fig. 5, Fig. 6).

Included in this study was a comparison of the effects of DNA size. Gilbert et al [15] found that the DNA size in commercial products ranged from between 100 and 200 bp to larger than 2,072 bp. The size of the pDNA derived from unprocessed SIS used in the current study was much larger (Supplemental Fig 1). As mentioned above, animals immunized with this material developed weak Th1 cytokine responses but were otherwise no different from the groups immunized with smaller pDNA remnants. Our study also showed that responses to pDNA from SIS before or after terminal sterilization were not significantly different.

A limitation of our study was that only 1 mammalian ECM species was examined. On the other hand, several mammalian sources are used for the production of ECM-based medical devices. Nonmammalian species, such as fish, are also used for ECM production, but these other sources were not tested in our study. Consequently, these results cannot be extrapolated to all ECM-based products, and further studies would be required to assess the potential immunological consequences of all ECM sources. An additional limitation is that we examined the xenogeneic reaction between porcine ECM and mice, not porcine ECM and humans. A technical limitation of the study was an inability to isolate DNA without potentially removing attached antigenic molecules, such as DNA-binding proteins. However, previous work examining the antibody response to mammalian DNA also used techniques to remove contaminating components from DNA [12], so this study follows the established precedent. Another limitation is the short time periods of follow-up; ECM-based materials may remain several months after implantation, possibly presenting remnant DNA during the whole degradation period. This work focused on the immediate immune response (5 weeks), whereas others have examined immune responses after an 8-week, second implantation of SIS [9] or 12 weeks after multiple implantations [23]. Additionally, surgical trauma, such as during an implantation, can induce or enhance an immune response, but multiple studies (for example, Choileain and Redmond [33]) suggest that surgical manipulation itself leads to an activated innate immune response but a suppressed adaptive immune response, perhaps both caused by IL-6. One effect is decreased antigen presentation function, with a likely decrease in antibody production. Thus, although we did not directly examine conditions of wound healing, we believe that our model likely resulted in greater levels of antibody production compared to the ECM DNA present in a surgical wound that could actually lead to suppressed adaptive immune responses. Lastly, we only examined antibody production and local cytokine response. As mentioned above, mtDNA could be present in the pDNA and could bind to TLR9 and activate neutrophils [34], another important part of the innate immune response, but this was not examined here.

So, why is this study important? Processing tissues into medical devices involves a delicate balance between removal of cellular debris and the retention of ECM structure and bioactive components (for review, see Jiang et al [35]). For example, some detergents can interfere with an ECM's collagen fiber organization and affect cellular growth on the ECM [36]. Decellularization can also prevent the formation of epithelial barriers, which is rescued by adding back stripped ECM components [37]. Lastly, enzymatically removing heparan sulfate (a growth factor binding proteoglycan) from lung ECM can prevent the organization and differentiation of endodermal cells [38]. These examples demonstrate that overprocessing ECM is potentially detrimental to the desired outcome; additional examples are given in reviews by Gilbert et al [39] and Crapo et al [40]. Although the extent of decellularization needs to be monitored, the choice of the "canary in the coal mine" is very important. Detection of DNA remnants has been recommended repeatedly, most likely because the assay is relatively simple; however, the tide seems to be turning [41]. The issue with focusing on removing DNA (which has low antigenicity) is the assumption that all other cellular remnants [41] and antigenic noncellular components are being equally removed [42]. But DNA removal techniques are less likely to remove other cellular remnants, which are likely to be more antigenic than DNA. For example, a recent report found that two decellularized ECMs, with similar levels of DNA but decellularized by different processes, caused differing immune responses after implantation in rats [43]. Wong and Griffiths [42] reviewed the literature describing the persistence of antigens in decellularized materials, which in some cases led to mortality. The results presented here support the monitoring of other antigens, such as α-Gal [44] and/or N-glycolylneuraminic acid (Neu5Gc) [45], because SIS-derived remnant DNA demonstrated low antigenicity. In fact, a Chinese pharmaceutical industry standard for the detection of remnant α-Gal antigens in medical devices exists [46], and similar assays could be developed.

In conclusion, our results indicate that SIS-derived DNA by itself, in dramatically higher than normal exposure levels, is essentially antigenically inert and becomes immunogenic only under the highly stimulatory and inflammatory conditions represented by inclusion of mBSA, IL-12, and IFA. Mammalian ECM-derived (porcine SIS) DNA fragments directly injected xenogeneically into mice did not stimulate a measurable, humoral immune response, but did induce an acute Th2-like healing response. Thus, this well-established immunological model predicts that ECM-derived DNA is not a significant source of Th1 immunogenicity for xenogeneic ECM biomaterials in humans. Although not yet planned, future work in this area could include better defining the human responses to xenogeneic DNA. The notion that DNA could possibly trigger an accommodation type response begs both short-term biological and long-term evolutionary questions. The possible antigens involved could also help influence the science of transplantation and not just the safety of biomaterials.

The following are the supplementary data related to this article.

## Author Contribution

Rae Ritchie: Conceptualization, Investigation, Methodology, Writing – original draft, Writing – review & editing. Sharon Salmon: Investigation, Formal analysis, Methodology. Michael Hiles: Conceptualization, Methodology, Writing – original draft, Writing – review & editing. Dennis Metzger: Conceptualization, Methodology, Formal analysis, Writing – original draft, Writing – review & editing.

## Conflict of Interest

Rae Ritchie and Michael Hiles are employees of Cook Biotech, Inc, and they both are inventors on patents surrounding the SIS technology. Sharon Salmon and Dennis Metzger have no conflicts of interest to report.

## Funding Source

Cook Biotech, Inc, funded this study, supplying test materials and paying for supplies.

## Ethics Approval

Experimental animal protocols were in accordance with the *Guide for the Care and Use of the Laboratory Animals* of the NIH [46]. All animal procedures were approved by the Institutional Animal Care and Use Committee at Albany Medical College (protocol number: 20-04001).
